# *catena*-Poly[[di-μ-chlorido-bis­{[6-methoxy-2-(4-methyl­phenyl­iminio­methyl)phenolato-κ^2^ *O*,*O*′]cadmium(II)}]-di-μ_2_-thio­cyanato-κ^2^ *N*:*S*;κ^2^ *S*:*N*]

**DOI:** 10.1107/S1600536808038099

**Published:** 2008-11-22

**Authors:** Hua-Qiong Li, Hui-Duo Xian, Jian-Feng Liu, Guo-Liang Zhao

**Affiliations:** a Zhejiang Key Laboratory for Reactive Chemistry on Solid Surfaces, Institute of Physical Chemistry, Zhejiang Normal University, Jinhua, Zhejiang 321004, People’s Republic of China, and, College of Chemistry and Life Science, Zhejiang Normal University, Jinhua 321004, Zhejiang, People’s Republic of China

## Abstract

The asymmetric unit of the title compound, [Cd_2_Cl_2_(NCS)_2_(C_15_H_15_NO_2_)_2_]_*n*_, contains the Schiff base 2-[(4-methyl­phenyl­imino)meth­yl]-6-methoxy­phenol (H*L*) ligand, one thio­cyanate and one chloride ligand coordinated to a cadmium centre. The cadmium centers are linked to each other *via* two thio­cyanate and two chloride bridges alternately, resulting in centrosymmetric zigzag chains running parallel to the *a* axis. The Cd^II^ coordination environment contains two Cl atoms, one thio­cyanate (SCN) S atom, one isothio­cyanate (NCS) N atom and two O atoms from the H*L* ligand. The Schiff base ligand is in the *trans* conformation.

## Related literature

For related literature regarding Schiff bases and their complexes, see: Mondal *et al.* (1999 ▶); Sen *et al.* (2006 ▶); Yi *et al.* (2004 ▶); Yu *et al.* (2007 ▶); Zhao *et al.* (2007 ▶); Zhou & Zhao (2007 ▶). For related structures, see: Ding *et al.* (2006 ▶); Suh *et al.* (2007 ▶).
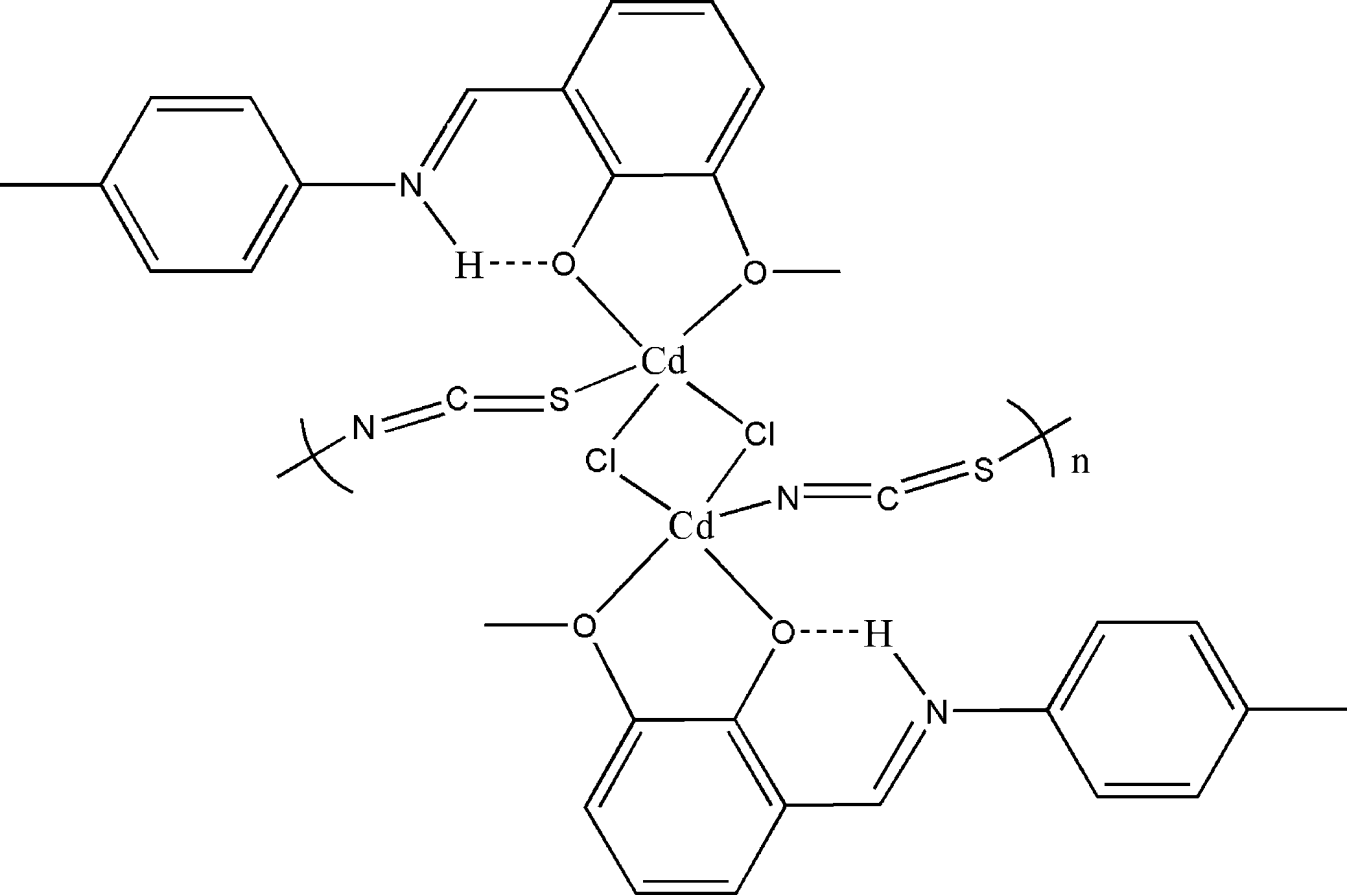


## Experimental

### 

#### Crystal data

[Cd_2_Cl_2_(NCS)_2_(C_15_H_15_NO_2_)_2_]
*M* *_r_* = 447.23
Triclinic, 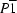

*a* = 9.0485 (2) Å
*b* = 9.7321 (2) Å
*c* = 10.6676 (3) Å
α = 71.518 (2)°
β = 77.444 (2)°
γ = 80.732 (2)°
*V* = 865.32 (4) Å^3^
*Z* = 2
Mo *K*α radiation
μ = 1.55 mm^−1^
*T* = 296 (2) K
0.27 × 0.11 × 0.08 mm

#### Data collection

Bruker APEXII diffractometer
Absorption correction: multi-scan (*SADABS*; Sheldrick, 1996 ▶) *T*_min_ = 0.82, *T*_max_ = 0.882
13032 measured reflections
3940 independent reflections
3225 reflections with *I* > 2σ(*I*)
*R*_int_ = 0.029

#### Refinement

*R*[*F* ^2^ > 2σ(*F* ^2^)] = 0.032
*wR*(*F* ^2^) = 0.081
*S* = 1.01
3940 reflections
208 parameters
H-atom parameters constrained
Δρ_max_ = 0.54 e Å^−3^
Δρ_min_ = −0.52 e Å^−3^

### 

Data collection: *APEX2* (Bruker, 2006 ▶); cell refinement: *SAINT* (Bruker, 2006 ▶); data reduction: *SAINT*; program(s) used to solve structure: *SHELXS97* (Sheldrick, 2008 ▶); program(s) used to refine structure: *SHELXL97* (Sheldrick, 2008 ▶); molecular graphics: *SHELXTL* (Sheldrick, 2008 ▶); software used to prepare material for publication: *SHELXL97*.

## Supplementary Material

Crystal structure: contains datablocks I, global. DOI: 10.1107/S1600536808038099/ez2146sup1.cif

Structure factors: contains datablocks I. DOI: 10.1107/S1600536808038099/ez2146Isup2.hkl

Additional supplementary materials:  crystallographic information; 3D view; checkCIF report

## Figures and Tables

**Fig. 1.**
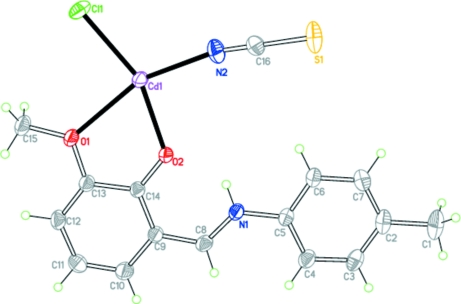
The coordination around the cadmium(II) center, showing the atom-labelling scheme. Displacement ellipsoids are drawn at the 30% probability level.

**Fig. 2.**
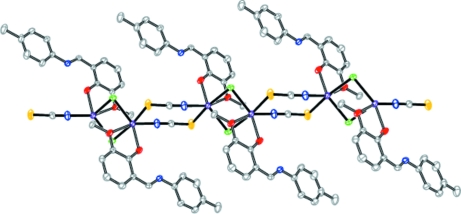
A perspective view of the title compound along the b axis. H atoms have been omitted for clarity.

## supplementary crystallographic information

### Comment

salen-type Schiff bases are capable of forming complexes with different coordination modes, with certain metal ions. Some of these compounds have promising applications in catalysis, enzyme models and optical and magnetic materials (Sen *et al.*, 2006). In addition, the unusual coordination modes of Schiff base ligands leads to unusual structures of the complexes. In previous articles (Zhou & Zhao, 2007; Yu *et al.*, 2007; Zhao *et al.*, 2007), we reported the synthesis and the ligating properties of the title Schiff base ligand, HL, derived from the condensation of *o*-vanillin and *p*-toluidine, to several transition and rare earth metals with different anions. In addition, many coordination polymers of one-, two-, and three-dimensional infinite frameworks involving cadmium(II) ions have been synthesized and studied due to their potential applications (Mondal *et al.*, 1999). Coordination polymers of cadmium(II) have been exploited using anionic ligands, *e.g.*, Cl^-^, Br^-^, I^-^, SCN^-^, N3^-^, SeCN^-^, *etc*., which are also an essential part of the coordination polyhedron, besides the organic ligand (Yi *et al.*, 2004). Here we decribe the synthesis and crystal structure of a new cadmium(II) complex (Figure 1), [Cd(HL)(SCN)Cl]_n_, involving the Schiff base HL.

As shown in Fig. 1 and 2, each Cd^II^ atom is hexacoordinated by two Cl atoms, one thiocyanate S atom, one isothiocyanate N atom and two O atoms from the Schiff base ligand, HL. The HL ligand is in the *trans* conformation. The geometry around the Cd^II^ atom is a distorted octahedron. Neighbouring octahedral Cd centres are bridged by, alternately, the SCN and NCS ligands and two Cl ligands to form alternating eight-membered Cd—S—C—N—Cd—S—C—N– and four-membered Cd—Cl—Cd—Cl- rings. These chains run parallel to the *a* axis. The Cd—S_SCN_ bond length is longer than the Cd—N_NCS_ distance [2.7096 (11) *versus* 2.2484 (26) Å], which, together with the bond angles, are similar to related compounds in the literatures (Suh *et al.*, 2007; Ding *et al.*, 2006).

### Experimental

First, the ligand was prepared by the direct solid-phase reaction of *o*-vanillin (10 mmol, 1.5251 g) and *p*-toluidine (10 mmol, 1.0700 g). The reactants were ground in an agate mortar. The color of the mixture changed from light yellow to orange. Then, for the preparation of the complex, a solution of CdCl_2_. 2.5H_2_O (1 mmol, 0.2931 g) and KSCN (0.1945 g, 2 mmol) in methanol (10 ml) was added to a methanol (30 ml) solution of the Schiff base ligand (2 mmol, 0.4826 g). Yellow crystals were obtained after 10 days.

### Refinement

The H atoms bonded to C and N atoms were positioned geometrically and refined using a riding model [aromatic C—H=0.93 Å, aliphatic C—H = 0.97 (2) Å, N—H=0.86 Å, *U*_iso_(H) = 1.2*U*_eq_(C,*N*)].

### Crystal data

**Table:**

[Cd_2_Cl_2_(NCS)_2_(C_15_H_15_NO_2_)_2_]	*Z* = 2
*M**_r_* = 447.23	*F*_000_ = 444
Triclinic, *P*1	*D*_x_ = 1.717 Mg m^−^^3^
Hall symbol: -P 1	Mo *K*α radiation λ = 0.71073 Å
*a* = 9.0485 (2) Å	Cell parameters from 4749 reflections
*b* = 9.7321 (2) Å	θ = 2.1–27.4º
*c* = 10.6676 (3) Å	µ = 1.55 mm^−^^1^
α = 71.518 (2)º	*T* = 296 (2) K
β = 77.444 (2)º	Block, red
γ = 80.732 (2)º	0.27 × 0.11 × 0.08 mm
*V* = 865.32 (4) Å^3^	

### Data collection

**Table:**

Bruker APEXII diffractometer	3940 independent reflections
Radiation source: fine-focus sealed tube	3225 reflections with *I* > 2σ(*I*)
Monochromator: graphite	*R*_int_ = 0.029
*T* = 296(2) K	θ_max_ = 27.4º
ω scans	θ_min_ = 2.1º
Absorption correction: multi-scan(SADABS; Sheldrick, 1996)	*h* = −11→11
*T*_min_ = 0.82, *T*_max_ = 0.882	*k* = −12→12
13032 measured reflections	*l* = −13→13

### Refinement

**Table:**

Refinement on *F*^2^	Secondary atom site location: difference Fourier map
Least-squares matrix: full	Hydrogen site location: inferred from neighbouring sites
*R*[*F*^2^ > 2σ(*F*^2^)] = 0.032	H-atom parameters constrained
*wR*(*F*^2^) = 0.081	*w* = 1/[σ^2^(*F*_o_^2^) + (0.0453*P*)^2^ + 0.1806*P*] where *P* = (*F*_o_^2^ + 2*F*_c_^2^)/3
*S* = 1.01	(Δ/σ)_max_ < 0.001
3940 reflections	Δρ_max_ = 0.54 e Å^−^^3^
208 parameters	Δρ_min_ = −0.52 e Å^−^^3^
Primary atom site location: structure-invariant direct methods	Extinction correction: none

### Special details

**Table:**

Geometry. All e.s.d.'s (except the e.s.d. in the dihedral angle between two l.s. planes) are estimated using the full covariance matrix. The cell e.s.d.'s are taken into account individually in the estimation of e.s.d.'s in distances, angles and torsion angles; correlations between e.s.d.'s in cell parameters are only used when they are defined by crystal symmetry. An approximate (isotropic) treatment of cell e.s.d.'s is used for estimating e.s.d.'s involving l.s. planes.
Refinement. Refinement of *F*^2^ against ALL reflections. The weighted *R*-factor *wR* and goodness of fit *S* are based on *F*^2^, conventional *R*-factors *R* are based on *F*, with *F* set to zero for negative *F*^2^. The threshold expression of *F*^2^ > σ(*F*^2^) is used only for calculating *R*-factors(gt) *etc*. and is not relevant to the choice of reflections for refinement. *R*-factors based on *F*^2^ are statistically about twice as large as those based on *F*, and *R*- factors based on ALL data will be even larger.

### Fractional atomic coordinates and isotropic or equivalent isotropic displacement parameters (Å^2^)

**Table:**

	*x*	*y*	*z*	*U*_iso_*/*U*_eq_	
Cd1	0.16960 (2)	0.06238 (2)	0.54427 (2)	0.04022 (10)	
Cl1	0.05869 (9)	0.10606 (10)	0.33628 (8)	0.0508 (2)	
O1	−0.0129 (2)	0.2707 (2)	0.5970 (2)	0.0479 (5)	
N1	0.2771 (3)	−0.0247 (3)	0.9681 (2)	0.0380 (5)	
H1D	0.2767	−0.0267	0.8882	0.046*	
C1	0.6806 (5)	−0.4746 (4)	1.2474 (5)	0.0786 (13)	
H1A	0.6777	−0.4648	1.3347	0.118*	
H1B	0.7825	−0.4685	1.1977	0.118*	
H1C	0.6482	−0.5672	1.2569	0.118*	
S1	0.64223 (10)	−0.27344 (10)	0.58734 (12)	0.0690 (3)	
O2	0.1735 (2)	0.0798 (2)	0.7460 (2)	0.0448 (5)	
C2	0.5752 (4)	−0.3537 (4)	1.1732 (4)	0.0549 (9)	
N2	0.3597 (3)	−0.1180 (3)	0.5586 (3)	0.0555 (7)	
C3	0.4944 (4)	−0.2504 (4)	1.2320 (4)	0.0538 (9)	
H3A	0.5066	−0.2555	1.3178	0.065*	
C4	0.3960 (4)	−0.1396 (4)	1.1679 (3)	0.0472 (8)	
H4A	0.3428	−0.0712	1.2097	0.057*	
C5	0.3782 (3)	−0.1326 (3)	1.0407 (3)	0.0385 (7)	
C6	0.4605 (4)	−0.2321 (4)	0.9780 (4)	0.0541 (9)	
H6A	0.4510	−0.2254	0.8911	0.065*	
C7	0.5572 (4)	−0.3416 (4)	1.0452 (4)	0.0645 (10)	
H7A	0.6116	−0.4091	1.0029	0.077*	
C8	0.1852 (3)	0.0767 (3)	1.0080 (3)	0.0405 (7)	
H8A	0.1835	0.0824	1.0937	0.049*	
C9	0.0880 (3)	0.1784 (3)	0.9282 (3)	0.0373 (6)	
C10	−0.0067 (4)	0.2857 (4)	0.9810 (3)	0.0543 (9)	
H10A	−0.0047	0.2876	1.0673	0.065*	
C11	−0.0998 (4)	0.3851 (4)	0.9058 (4)	0.0604 (10)	
H11A	−0.1604	0.4560	0.9404	0.073*	
C12	−0.1062 (3)	0.3829 (3)	0.7766 (3)	0.0464 (8)	
H12A	−0.1718	0.4514	0.7267	0.056*	
C13	−0.0171 (3)	0.2811 (3)	0.7232 (3)	0.0369 (6)	
C14	0.0858 (3)	0.1752 (3)	0.7971 (3)	0.0334 (6)	
C15	−0.1276 (4)	0.3549 (4)	0.5233 (3)	0.0516 (8)	
H15A	−0.1801	0.4271	0.5654	0.077*	
H15B	−0.1987	0.2921	0.5221	0.077*	
H15C	−0.0812	0.4019	0.4330	0.077*	
C16	0.4777 (4)	−0.1803 (3)	0.5696 (3)	0.0441 (7)	

### Atomic displacement parameters (Å^2^)

**Table:**

	*U*^11^	*U*^22^	*U*^33^	*U*^12^	*U*^13^	*U*^23^
Cd1	0.03390 (14)	0.05451 (16)	0.03464 (15)	0.00929 (10)	−0.01210 (9)	−0.01906 (11)
Cl1	0.0445 (4)	0.0800 (6)	0.0284 (4)	−0.0077 (4)	−0.0083 (3)	−0.0144 (4)
O1	0.0485 (12)	0.0591 (13)	0.0372 (12)	0.0207 (10)	−0.0209 (10)	−0.0196 (10)
N1	0.0383 (13)	0.0445 (13)	0.0308 (14)	0.0031 (11)	−0.0115 (10)	−0.0104 (11)
C1	0.056 (2)	0.062 (2)	0.100 (3)	−0.0001 (19)	−0.035 (2)	0.012 (2)
S1	0.0392 (5)	0.0516 (5)	0.0980 (8)	0.0070 (4)	−0.0190 (5)	0.0023 (5)
O2	0.0492 (13)	0.0502 (12)	0.0392 (12)	0.0204 (10)	−0.0211 (10)	−0.0226 (10)
C2	0.0390 (18)	0.0470 (19)	0.068 (3)	−0.0045 (15)	−0.0191 (17)	0.0037 (17)
N2	0.0361 (15)	0.0505 (16)	0.076 (2)	0.0055 (13)	−0.0112 (14)	−0.0172 (15)
C3	0.052 (2)	0.063 (2)	0.040 (2)	−0.0058 (17)	−0.0179 (16)	0.0018 (16)
C4	0.0464 (18)	0.0571 (19)	0.0355 (18)	0.0009 (15)	−0.0106 (14)	−0.0105 (15)
C5	0.0341 (15)	0.0419 (16)	0.0375 (17)	−0.0010 (12)	−0.0119 (13)	−0.0066 (13)
C6	0.052 (2)	0.061 (2)	0.051 (2)	0.0156 (16)	−0.0234 (17)	−0.0204 (17)
C7	0.057 (2)	0.062 (2)	0.079 (3)	0.0174 (18)	−0.025 (2)	−0.030 (2)
C8	0.0395 (17)	0.0526 (18)	0.0284 (16)	−0.0012 (14)	−0.0054 (13)	−0.0125 (13)
C9	0.0336 (15)	0.0459 (16)	0.0314 (16)	0.0038 (13)	−0.0064 (12)	−0.0132 (13)
C10	0.056 (2)	0.069 (2)	0.0392 (19)	0.0146 (17)	−0.0108 (16)	−0.0249 (17)
C11	0.058 (2)	0.069 (2)	0.058 (2)	0.0254 (18)	−0.0117 (18)	−0.0367 (19)
C12	0.0386 (17)	0.0500 (18)	0.0453 (19)	0.0127 (14)	−0.0117 (14)	−0.0123 (15)
C13	0.0337 (15)	0.0425 (16)	0.0338 (16)	0.0011 (12)	−0.0088 (12)	−0.0106 (13)
C14	0.0290 (14)	0.0377 (15)	0.0323 (16)	0.0005 (11)	−0.0056 (11)	−0.0104 (12)
C15	0.0463 (19)	0.062 (2)	0.044 (2)	0.0107 (16)	−0.0222 (15)	−0.0110 (16)
C16	0.0392 (17)	0.0434 (17)	0.048 (2)	−0.0021 (14)	−0.0039 (14)	−0.0144 (14)

### Geometric parameters (Å, °)

**Table:**

Cd1—O2	2.2191 (19)	C3—C4	1.383 (4)
Cd1—N2	2.244 (3)	C3—H3A	0.9300
Cd1—Cl1	2.5187 (8)	C4—C5	1.381 (4)
Cd1—O1	2.529 (2)	C4—H4A	0.9300
Cd1—Cl1^i^	2.6833 (9)	C5—C6	1.379 (4)
Cd1—S1^ii^	2.7107 (10)	C6—C7	1.380 (5)
Cl1—Cd1^i^	2.6833 (9)	C6—H6A	0.9300
O1—C13	1.373 (3)	C7—H7A	0.9300
O1—C15	1.428 (4)	C8—C9	1.410 (4)
N1—C8	1.303 (4)	C8—H8A	0.9300
N1—C5	1.421 (4)	C9—C14	1.413 (4)
N1—H1D	0.8600	C9—C10	1.420 (4)
C1—C2	1.515 (5)	C10—C11	1.352 (5)
C1—H1A	0.9600	C10—H10A	0.9300
C1—H1B	0.9600	C11—C12	1.400 (5)
C1—H1C	0.9600	C11—H11A	0.9300
S1—C16	1.629 (3)	C12—C13	1.362 (4)
S1—Cd1^ii^	2.7107 (10)	C12—H12A	0.9300
O2—C14	1.299 (3)	C13—C14	1.430 (4)
C2—C7	1.376 (5)	C15—H15A	0.9600
C2—C3	1.379 (5)	C15—H15B	0.9600
N2—C16	1.150 (4)	C15—H15C	0.9600
			
O2—Cd1—N2	92.93 (9)	C3—C4—H4A	120.7
O2—Cd1—Cl1	155.30 (6)	C6—C5—C4	120.3 (3)
N2—Cd1—Cl1	110.91 (8)	C6—C5—N1	117.0 (3)
O2—Cd1—O1	67.95 (7)	C4—C5—N1	122.7 (3)
N2—Cd1—O1	160.37 (10)	C5—C6—C7	119.4 (3)
Cl1—Cd1—O1	88.62 (5)	C5—C6—H6A	120.3
O2—Cd1—Cl1^i^	86.93 (6)	C7—C6—H6A	120.3
N2—Cd1—Cl1^i^	96.98 (7)	C2—C7—C6	121.8 (3)
Cl1—Cd1—Cl1^i^	83.92 (3)	C2—C7—H7A	119.1
O1—Cd1—Cl1^i^	86.77 (6)	C6—C7—H7A	119.1
O2—Cd1—S1^ii^	94.23 (6)	N1—C8—C9	123.5 (3)
N2—Cd1—S1^ii^	93.69 (8)	N1—C8—H8A	118.3
Cl1—Cd1—S1^ii^	90.71 (3)	C9—C8—H8A	118.3
O1—Cd1—S1^ii^	83.73 (6)	C8—C9—C14	120.8 (2)
Cl1^i^—Cd1—S1^ii^	169.20 (3)	C8—C9—C10	118.9 (3)
Cd1—Cl1—Cd1^i^	96.08 (3)	C14—C9—C10	120.3 (3)
C13—O1—C15	118.3 (2)	C11—C10—C9	119.9 (3)
C13—O1—Cd1	113.42 (16)	C11—C10—H10A	120.0
C15—O1—Cd1	126.96 (18)	C9—C10—H10A	120.0
C8—N1—C5	127.9 (3)	C10—C11—C12	121.0 (3)
C8—N1—H1D	116.1	C10—C11—H11A	119.5
C5—N1—H1D	116.1	C12—C11—H11A	119.5
C2—C1—H1A	109.5	C13—C12—C11	120.5 (3)
C2—C1—H1B	109.5	C13—C12—H12A	119.7
H1A—C1—H1B	109.5	C11—C12—H12A	119.7
C2—C1—H1C	109.5	C12—C13—O1	125.2 (3)
H1A—C1—H1C	109.5	C12—C13—C14	121.0 (3)
H1B—C1—H1C	109.5	O1—C13—C14	113.9 (2)
C16—S1—Cd1^ii^	100.35 (12)	O2—C14—C9	121.3 (3)
C14—O2—Cd1	123.29 (18)	O2—C14—C13	121.4 (3)
C7—C2—C3	117.5 (3)	C9—C14—C13	117.3 (2)
C7—C2—C1	121.8 (4)	O1—C15—H15A	109.5
C3—C2—C1	120.7 (4)	O1—C15—H15B	109.5
C16—N2—Cd1	160.6 (3)	H15A—C15—H15B	109.5
C2—C3—C4	122.3 (3)	O1—C15—H15C	109.5
C2—C3—H3A	118.8	H15A—C15—H15C	109.5
C4—C3—H3A	118.8	H15B—C15—H15C	109.5
C5—C4—C3	118.6 (3)	N2—C16—S1	178.1 (3)
C5—C4—H4A	120.7		
			
O2—Cd1—Cl1—Cd1^i^	−68.87 (15)	C8—N1—C5—C6	177.7 (3)
N2—Cd1—Cl1—Cd1^i^	95.17 (8)	C8—N1—C5—C4	−2.6 (5)
O1—Cd1—Cl1—Cd1^i^	−86.90 (6)	C4—C5—C6—C7	2.1 (5)
Cl1^i^—Cd1—Cl1—Cd1^i^	0.0	N1—C5—C6—C7	−178.2 (3)
S1^ii^—Cd1—Cl1—Cd1^i^	−170.61 (3)	C3—C2—C7—C6	−0.9 (6)
O2—Cd1—O1—C13	−2.23 (18)	C1—C2—C7—C6	179.6 (3)
N2—Cd1—O1—C13	−16.0 (4)	C5—C6—C7—C2	−0.7 (6)
Cl1—Cd1—O1—C13	169.75 (19)	C5—N1—C8—C9	−179.5 (3)
Cl1^i^—Cd1—O1—C13	85.76 (19)	N1—C8—C9—C14	−0.1 (5)
S1^ii^—Cd1—O1—C13	−99.38 (19)	N1—C8—C9—C10	−179.3 (3)
O2—Cd1—O1—C15	−169.0 (3)	C8—C9—C10—C11	179.4 (3)
N2—Cd1—O1—C15	177.2 (3)	C14—C9—C10—C11	0.2 (5)
Cl1—Cd1—O1—C15	3.0 (2)	C9—C10—C11—C12	1.0 (6)
Cl1^i^—Cd1—O1—C15	−81.0 (2)	C10—C11—C12—C13	−0.8 (6)
S1^ii^—Cd1—O1—C15	93.8 (2)	C11—C12—C13—O1	−178.9 (3)
N2—Cd1—O2—C14	177.8 (2)	C11—C12—C13—C14	−0.6 (5)
Cl1—Cd1—O2—C14	−17.1 (3)	C15—O1—C13—C12	−11.6 (5)
O1—Cd1—O2—C14	2.4 (2)	Cd1—O1—C13—C12	−179.6 (3)
Cl1^i^—Cd1—O2—C14	−85.3 (2)	C15—O1—C13—C14	169.9 (3)
S1^ii^—Cd1—O2—C14	83.9 (2)	Cd1—O1—C13—C14	1.9 (3)
O2—Cd1—N2—C16	−59.5 (9)	Cd1—O2—C14—C9	177.4 (2)
Cl1—Cd1—N2—C16	127.1 (9)	Cd1—O2—C14—C13	−2.4 (4)
O1—Cd1—N2—C16	−46.8 (10)	C8—C9—C14—O2	−0.5 (4)
Cl1^i^—Cd1—N2—C16	−146.8 (9)	C10—C9—C14—O2	178.7 (3)
S1^ii^—Cd1—N2—C16	34.9 (9)	C8—C9—C14—C13	179.3 (3)
C7—C2—C3—C4	1.3 (5)	C10—C9—C14—C13	−1.4 (4)
C1—C2—C3—C4	−179.2 (3)	C12—C13—C14—O2	−178.5 (3)
C2—C3—C4—C5	0.1 (5)	O1—C13—C14—O2	0.0 (4)
C3—C4—C5—C6	−1.8 (5)	C12—C13—C14—C9	1.7 (4)
C3—C4—C5—N1	178.5 (3)	O1—C13—C14—C9	−179.8 (3)

Symmetry codes: (i) −*x*, −*y*, −*z*+1; (ii) −*x*+1, −*y*, −*z*+1.
